# Development of a real-time PCR method for the specific detection of the novel pear pathogen *Erwinia uzenensis*

**DOI:** 10.1371/journal.pone.0219487

**Published:** 2019-07-10

**Authors:** Maria C. Holeva, Félix Morán, Giuseppe Scuderi, Asier González, María M. López, Pablo Llop

**Affiliations:** 1 Laboratory of Bacteriology, Department of Plant Pathology, Benaki Phytopathological Institute, Kifissia, Attica, Greece; 2 Laboratory of Bacteriology, Department of Plant Pathology, Instituto Valenciano de Investigaciones Agrarias (IVIA), Moncada, Spain; 3 Laboratory of Biotechnology, Department of Sustainable agriculture, biodiversity and food security, Euro-Mediterranean Institute of Science and Technology (IEMEST), Palermo, Italy; Defense Threat Reduction Agency, UNITED STATES

## Abstract

*Erwinia uzenensis* is a plant-pathogenic bacterium, recently described in Japan, which infects pear trees, causing the ‘bacterial black shoot disease of European pear’ (BBSDP). Like other *Erwinia* pear pathogens, *E*. *uzenensis* causes damp, black lesions on young shoots resembling those of *E*. *amylovora*, but not blossom blight, fruitlet blight or wilting of the shoot tip. The distribution of *E*. *uzenensis* seems restricted to the country where it was reported up to now, but it may spread to other countries and affect new hosts, as is the current situation with *E*. *piriflorinigrans* and *E*. *pyrifoliae*. Fast and accurate detection systems for this new pathogen are needed to study its biology and to identify it on pear or other hosts. We report here the development of a specific and sensitive detection protocol based on a real-time PCR with a TaqMan probe for *E*. *uzenensis*, and its evaluation. In sensitivity assays, the detection threshold of this protocol was 10^1^ cfu ml^-1^ on pure bacterial cultures and 10^2^–10^3^ cfu ml^-1^ on spiked plant material. The specificity of the protocol was evaluated against *E*. *uzenensis* and 46 strains of pear-associated *Erwinia* species different to *E*. *uzenensis*. No cross-reaction with the non-target bacterial species or the loss of sensitivity were observed. This specific and sensitive diagnostic tool may reveal a wider distribution and host range of *E*. *uzenensis* initially considered restricted to a region and will expand our knowledge of the life cycle and environmental preferences of this pathogen.

## Introduction

*Erwinia uzenensis* is a Gram-negative bacterium, non-spore-forming, facultatively anaerobic, pathogenic on European pear (*Pyrus communis*), causing damp, black lesions on young shoots, which occasionally extend from the shoot through the petioles to the main vein of leaves [1]. The disease has been named ‘bacterial black shoot disease of European pear’ (BBSDP) and has been recorded only in Japan and only in pear trees [1, 2]. The symptoms of BBSDP generally resemble those caused on shoots by other necrotrophic *Erwinia* species pathogenic to pear (*E*. *amylovora* and *E*. *pyrifoliae*), except they do not include blossom blight, fruitlet blight and wilting of the shoot tip, thus rendering the impact of the disease less severe than that of the other *Erwinia* pathogens [1]. However, due to its relatively recent first description in Japan, the epidemiological characteristics of this new disease are poorly studied and understood. Recent records of *E*. *piriflorinigrans* in new hosts in Spain [3] or in new areas in Switzerland (Smits, T., personal communication), as well as the discovery of *E*. *pyrifoliae* in strawberry plants in The Netherlands [4] suggest that the likelihood of spread of *E*. *uzenensis* and subsequent yield losses due to its infections could be higher than it is currently anticipated. Consequently, despite its restricted range of symptoms, hosts and geographical distribution, *E*. *uzenensis* could represent a risk for pear production, although with high uncertainty. Furthermore, the differentiation of *E*. *uzenensis* from the other pear-associated *Erwinia* species, including the pathogenic species *E*. *amylovora*, *E*. *pyrifoliae*, *E*. *piriflorinigrans* and the non-pathogenic rosaceous epiphytes *E*. *tasmaniensis*, *E*. *billingiae* and *E*. *gerundensis* [5] is of critical importance for exporting commodities, since phytosanitary requirements of importing countries often include certification of absence of certain *Erwinia* plant pathogens. Especially in the EU, *E*. *amylovora* constitutes a regulated harmful organism listed in the phytosanitary legislation [6, 7], and thus its occurrence has severe negative impact not only due to the loss of yield and even the plant capital but also due to the loss of export markets and the imposition of official quarantine measures, including costly eradication procedures. Additionally, the occurrence of mixed infections of related pathogens, as described for host plants affected by both *E*. *amylovora* and *E*. *piriflorinigrans* [3], should be taken into account when examining disease outbreaks.

Recently, Matsuura *et al*. [2] described *E*. *uzenensis* as a novel species of the genus *Erwinia*, and provided data on phenotypic characteristics, *viz*. growth factor requirements, fatty acid content and metabolic profile based on *β*-glucosidase activity, *β*-galactosidase activity, and acid production, to differentiate the novel species from related species of the genus. Additionally, phylogenetic analyses based on the 16S rRNA, *gyrB* and *rpoD* genes and genetic characteristics, *i*.*e*. DNA-DNA hybridization and DNA G+C content further differentiated the novel species [2]. Mizuno *et al*. [1] reported that morphological characteristics of *E*. *uzenensis* colonies on commonly used media for isolation of pear bacterial pathogens resembled those of *E*. *amylovora* or *E*. *pyrifoliae*. Besides, PCR with the primer set EprpoD developed previously could not distinguish between *E*. *uzenensis*, *E*. *pyrifoliae*, and *E*. *amylovora* biovar 4 [1]. Moreover, no species-specific antiserum has been reported for *E*. *uzenensis* that could be used in serological diagnostic tests. As a result, currently available diagnostic methods could fail to detect or lead to misidentification of this new bacterial pathogen of pear, as it shares many morphological, biochemical, metabolic and genetic characteristics with the above mentioned pathogenic and non-pathogenic pear-associated *Erwinia* species. For the above reasons, a diagnostic methodology for rapid and accurate detection and identification of *E*. *uzenensis* is urgently needed for prompt clarification of the status of this pathogen, effective management of BBSDP, unambiguous differentiation of *E*. *uzenensis* from the other *Erwinia* species associated with pear and understanding the synergistic effect of these pathogens in the progression of the outbreaks.

The aim of this study was to develop a real-time PCR protocol targeting the rRNA operon of *E*. *uzenensis*. To this end, primers and a TaqMan probe for the PCR protocol were designed, taking especially into account the need to distinguish *E*. *uzenensis* from other pathogenic and non-pathogenic *Erwinia* species associated with pear. The specificity and sensitivity of the developed TaqMan PCR protocol were also evaluated. The use of such a method would greatly enhance the efficiency of survey programs and improve our knowledge in relation to the life cycle of *E*. *uzenensis* and its interaction with the other pear-associated bacteria in the natural environment.

## Materials and methods

### Bacterial strains

Bacterial strains employed in this study are listed in Table 1. The strains of *E*. *uzenensis* (LMG 25842, LMG 25844 and LMG 25845), obtained from the Belgian Coordinated Collection of Micro-Organisms (BCCM^TM^), as well as all the other *Erwinia* strains employed in specificity and sensitivity assays, were grown on King’s B medium [8] at 26°C for 48 h. *Escherichia coli* strain JM109 used for cloning experiments was incubated at 37°C on LB [9] broth or agar plates.

**Table 1:** Bacterial strains used in this study.

Bacterial strain^a^	Host	Origin	Reference
*E*. *uzenensis*			
LMG 25842	*Pyrus communis*	Japan	[1, 2]
LMG 25844	*P*. *communis*	Japan	[1, 2]
LMG 25845	*P*. *communis*	Japan	[1]
*E*. *amylovora*			
CFBP 1430	*Crataegus oxyacantha*	France	[26]
ARI CGJ-2	*Malus* sp.	Serbia	[27]
ARI BC3	*Malus* sp.	Serbia	[27]
SL E-70	*Cotoneaster* sp.	Ireland	[29]
UPN 527	*Malus* sp.	Spain	[28]
IVIA 1554	*Crataegus* sp.	Spain	[28]
IVIA 1596	*Pyrus* sp.	Spain	[28]
IVIA 1739–1	*Malus* sp.	Spain	[28]
IVIA 1951–8	*Pyracantha* sp.	Spain	[28]
NIB 311	*Pyrus* sp.	Slovenia	[28]
DPP Ea PO 394	NA^b^	Poland	NA
DPP Ea PO 632	NA	Poland	NA
DPP Ea PO 659	NA	Poland	NA
BBA KG 6–45	*Cydonia* sp.	France	[28]
BBA KG 9–43	*Cotoneaster* sp.	France	[28]
BBA KG 9–7	*Pyrus* sp.	France	[28]
BBA KG 250	*Malus* sp.	Germany	[28]
BBA KG 1/79	*Cotoneaster* sp.	Germany	[28]
AGES E.a. 1	*Malus* sp.	Hungary	[28]
AGES E.a.17	*Cydonia* sp.	Hungary	[28]
AGES E.a. 26	*Pyrus* sp.	Hungary	[28]
AGES E.a. 28	*Crataegus* sp.	Hungary	[28]
AGES 295/93	*Cotoneaster salicifolius*	Austria	[28]
AGES 483/98	*Cotoneaster* sp.	Austria	[28]
AGES 1186/00	*Sorbus* sp.	Austria	[28]
LNPV 1585	*Cotoneaster* sp.	France	[28]
LNPV 1626	*Pyrus* sp.	France	[28]
LNPV 1709	*Cotoneaster* sp.	France	[28]
LNPV 1879	*Cotoneaster* sp.	France	[3]
CFBP 3012	*Crataegus monogyna*	Belgium	[30]
CFBP 3098	*Malus domestica*	Israel	[31]
BPIC 909	*Pyrus* sp.	Greece	[28]
BPIC 913	*Pyrus* sp.	Greece	[28]
BPIC 917	*Cydonia* sp.	Greece	[28]
BPIC 1041	*Cydonia* sp.	Greece	[28]
SL 2156	*Cotoneaster* sp.	Ireland	[28]
SL 2160	*Sorbus* sp.	Ireland	[28]
SL 2164	*Sorbus* sp.	Ireland	[28]
*E*. *pyrifoliae*			
CFBP 4172	*P*. *pyrifolia*	South Korea	[32]
CFBP 4243	*P*. *pyrifolia*	South Korea	[32]
*E*. *piriflorinigrans*			
CFBP 5881	*Pyrus* sp.	Spain	[33]
CFBP 5888	*Pyrus* sp.	Spain	[33]
*E*. *tasmaniensis*			
NCPPB 4357	*Malus* sp.	Australia	[34]
NCPPB 4358	*Pyrus* sp.	Australia	[34]
*E*. *billingiae*			
IVIA 3571–1	*Malus* sp.	Spain	[3]
LMG 2624	*Crataegus* sp.	France	[35]

^a^The acronyms in the names of the bacterial strains stand for:

ARI: Agricultural Research Institute SERBIA—Pesticide and Environmental Research Centre

Belgrade-Zemun. Serbia

BBA: Federal Biological Institute for Agriculture and Forestry. Dossenheim, Germany

BPIC: Benaki Phytopathological Institute Collection, Athens, Greece

CFBP: Collection Française de Bactéries Phytopathogènes, INRA, Angers, France

IVIA: Instituto Valenciano de Investigaciones Agrarias Collection, Moncada, Spain

LMG: Bacterial Collection of the Laboratory of Microbiology, Rijksuniversiteit, Ghent, Belgium

LNPV: Laboratoire National de la Protection des Végétaux, Beaucouzé, France

AGES: Austrian Agency for Health and Food Safety, Wien, Austria. MK: strains kindly provided by Dr. Marianne Keck

NCPPB: National Collection of Plant Pathogenic Bacteria, York, United Kingdom

NIB: National Institute of Biology, Ljubljana, Slovenia

SL: State Laboratory, Dublin, Ireland

UPN: Universidad Pública de Navarra, Pamplona, Spain

DPP: Department of Plant Protection collection, Institute of Horticulture, Pomology Divison, Skiernewice, Poland

^b^NA: data not available

### Sequencing of the *E*. *uzenensis* ribosomal operon

Sequence determination of the ribosomal operon of *E*. *uzenensis* was performed with PCR amplicons obtained by applying the 16SrRNA gene universal primer FGPS6 and the 23SrRNA gene reverse primer FGPL132' [10] (Table 2) on the three *E*. *uzenensis* strains employed in this study (Table 1). Briefly, 5 μl of bacterial suspension of 10^6^-10^7^cfu ml^-1^ were added in a 50 μl reaction mixture volume containing 1 U Taq DNA polymerase (Biotools B&M Labs S.A., Madrid, Spain), 0.1 μM of each primer, 0.2 mM of each of four dNTPs and 3 mM MgCl_2_. The mixture was subjected to an initial denaturation step of 3 min at 94°C, followed by 40 cycles of 35 s at 94°C, 1 min at 55°C and 2 min at 72°C. The amplification products obtained were visualized by electrophoresis on 1% (w/v) agarose gel in 0.5× TAE buffer (40 mM Tris base, 40 mM acetic acid, 1 mM EDTA) and ethidium bromide staining. A 1-kb DNA molecular weight ladder (Invitrogen, USA) was used in gel electrophoresis.

**Table 2:** Oligonucleotide PCR primers employed.

Primers	5'-3' Sequence	Reference
FGPS6	GGAGAGTTAGATCTTGGCTCAG	[10]
FGPL132'	CCGGGTTTCCCCATTCGG	
M13F	CGCCAGGGTTTTCCCAGTCACGAC	Promega, USA
M13R	AGCGGATAACAATTTCACACAGGA	
1389R	ACGGGCGGTGTGTACAAG	[11]
R16-1F	CTTGTACACACCGCCCGTCA	[12]
UzeF	TGCTGCGTTATGTTCTCGGC	This work
UzeR	AAACCTTTGTCTACCACTCA	This work
UzeP	FAM-ATTACATTTT/ZEN/CAGTTATCAAAAAGAA-IABkFQ	This work

**Fig 1:**
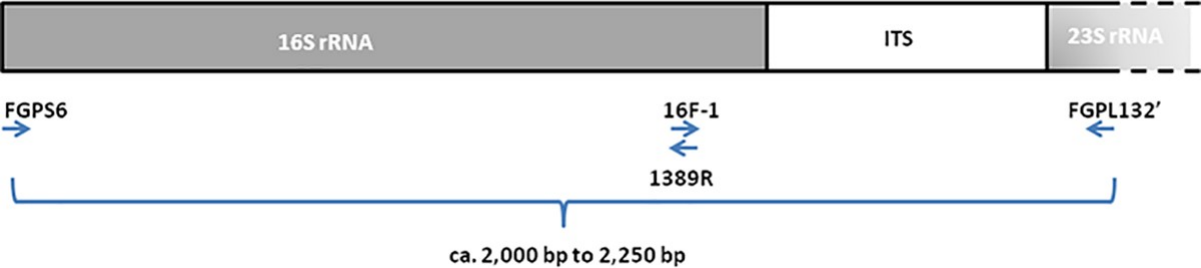
Primer and probe positions in the bacterial ribosomal operon. Priming directions are indicated by arrows. The PCR products generated by the primer set FGPS6/FGPL132’ (ca. 2000 bp to 2250 bp) were cloned into the pGEM-T-Easy vector (Promega, USA) and sequenced with: a) the primers M13F and M13R (not shown in the figure), annealing to either side of the vector’s cloning site; b) the primers 16F-1 and 1389R, annealing internally to the cloned insert. The positions of the newly designed real-time PCR primer set UzeF/UzeR and probe UzeP are also indicated. The figure is not drawn to scale.

For all three strains, the FGPS6/FGPL132' PCR resulted in two bands migrating closely together in the gel (described as ‘H’ and ‘L’ bands in the Results section); for each strain, the two bands were excised together from the gel using the UltraClean 15 DNA purification kit (MoBio Laboratories, USA), ligated with pGEM-T-Easy vector (Promega, USA) and transformed into *E*. *coli* JM109 chemically competent cells, following the instructions of vector’s supplier. Colony PCR with the universal primers M13F and M13R (Promega, USA) (Table 2) identified *E*. *coli* clones with ligated inserts corresponding to the ‘H’ or ‘L’ band. From the obtained *E*. *coli* clones, recombinant plasmid DNA was extracted using the REAL miniprep turbo kit (REAL, Spain). Sequencing of the ligated inserts was performed by IBMCP (Univ. Pol. de Valencia-CSIC, Spain) using the universal primers M13F and M13R (Promega, USA) that anneal to either side of the vector’s cloning site. The primers 1389R [11] and R16-1 [12] (Table 2), which anneal internally to the inserts, were also used for sequencing, to achieve coverage of the whole length of the cloned inserts. All primers used in this study are listed in Table 2 and their position at the rRNA operon is shown in Fig 1. The sequences obtained were assembled using the software Vector NTI 8 (Life Technologies, USA) and the consensus sequences of the inserts corresponding to the ‘H’ or ‘L’ band for the three *E*. *uzenensis* strains under study were obtained. These sequences were then subjected to BLASTn analysis against the GenBank non-redundant nucleotide database (NCBI).

### Structural annotation of sequenced ribosomal region

The structural organization of the sequenced region of the rRNA operon of each of the three *E*. *uzenensis* strains was inferred by BLASTn analysis against the GenBank non-redundant nucleotide database (NCBI), as well as by applying the software RNAmmer [13] and tRNA scan-SE [14]. RNAmmer predicts rRNA genes (5s, 16s, and 23s), while the tRNAscan-SE predicts transfer RNA genes (tRNAs) in the 16-23S rRNA internal transcribed spacer (ITS). They were applied using default values, after setting the organism type to ‘bacteria’.

### Design of real-time PCR primers and probe

**Fig 2:**
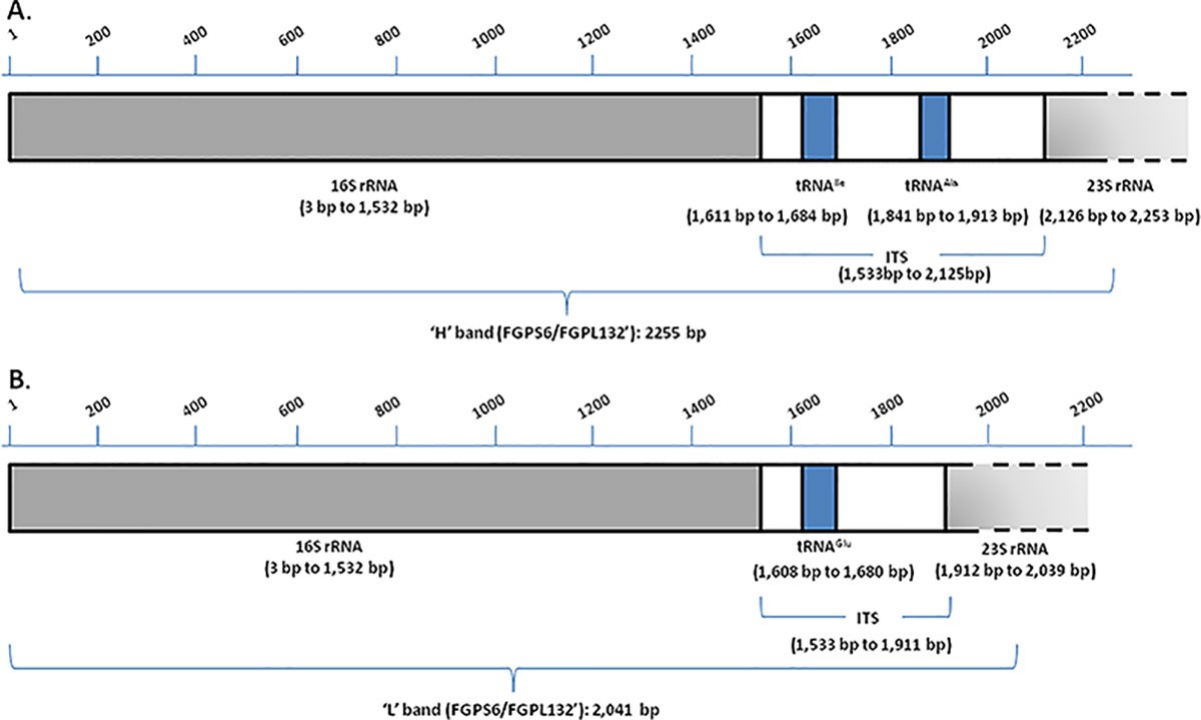
Schematic representation of the two structural organization patterns predicted for the sequenced 16S-ITS-23S rRNA region in all three *E*. *uzenensis* strains. A) Pattern corresponding to PCR bands with the higher molecular weight (‘H’); B) Pattern corresponding to PCR bands with the lower molecular weight (‘L’). Numbers in parentheses indicate the position of the 16S rRNA and 23S rRNA genes, of the tRNA molecules and of the ITS region in the sequence of the FGPS6/FGPL132’ PCR amplicons.

**Fig 3:**
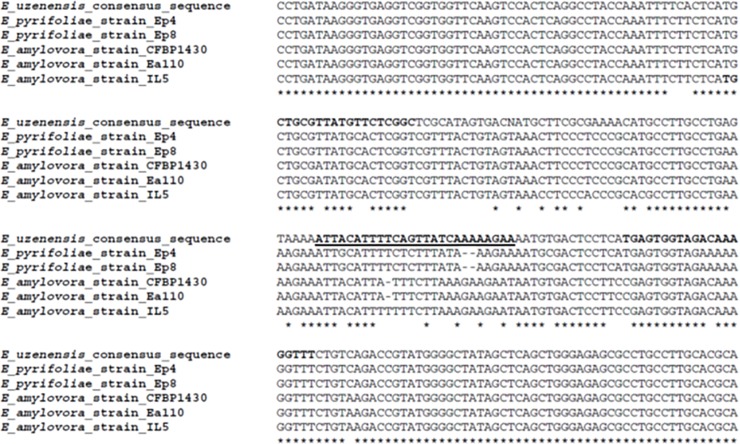
Multiple alignment analysis of the ITS sequences. Using CLUSTALW, the consensus ITS sequence of the ‘H’ bands of the three *E*. *uzenensis* strains and those ITS sequences of five other pathogenic *Erwinia* strains (*E*. *pyrifoliae* strains: Ep4 and Ep8; *E*. *amylovora* strains: CFBP1430, Ea110, and EaIL5) obtained from GenBank were aligned. The position of the newly designed primers (in bold) and probe (bold and underlined) for the real-time PCR protocol are shown.

In order to find a sufficiently variable area to design primers and probe to discriminate *E*. *uzenensis* from the other *Erwinia* species, the consensus sequence of the ‘H’ bands along the ITS region of the three *E*. *uzenensis* strains was compared using software CLUSTALW [15] with those of other *Erwinia* species pathogenic to pear, namely *E*. *amylovora* (strains: CFBP1430, Ea110 and EaIL5) and *E*. *pyrifoliae* (strains: Ep8 and Ep4), retrieved from GenBank. A pair of primers (UzeF, UzeR) was designed using the software Vector NTI 8 (Life Technologies, USA) to anneal to the region of the ITS that showed the highest variability between the strains analyzed, in order to achieve the best specificity (Figs 2 and 3). A TaqMan probe (UzeP) was also designed inside the selected region. This probe includes the ZEN Internal Quencher (Integrated DNA Technologies, Inc., USA) that enables less background and increased signal (better sensitivity) in the PCR assays (Table 2).

### Real-time PCR conditions

The real-time PCR assays were carried out in a StepOne thermocycler (Applied Biosystems, ThermoFisher, Waltham, MA, USA). The template used was a culture suspension of 10^8^ cfu ml^-1^, and dilution series from 10^5^ to 10^1^ cfu ml^-1^ of the *E*. *uzenensis* LMG25844 strain (Table 1). The amplification was performed in a 25 μl reaction volume containing 5 μl template (sample), 12.5 μl 2x reaction mix (Quantimix Easy Probes kit, Biotools B&M Labs S.A., Madrid, Spain), 0.2 μM of each primer (UzeF-UzeR) and 0.1 μM of TaqMan probe (UzeP). The amplification conditions included an initial denaturation step at 95°C for 10 min, followed by 40 cycles, each one consisting of 15 sec at 95°C and 30 sec at 60°C.

### Specificity and sensitivity assays

The specificity of the primers and probe designed in this work to detect *E*. *uzenensis* was first evaluated by BLASTn search of their sequences against GenBank database. Additionally, specificity assays using the developed real-time PCR protocol were performed on bacterial suspensions of 38 strains of *E*. *amylovora*, 2 strains of *E*. *piriflorinigrans*, 2 strains of *E*. *pyrifoliae* and 4 strains of non-pathogenic *Erwinia* species, all listed in Table 1. The analyses were performed twice with all the listed bacterial strains.

The analysis was extended to include real-time PCR detection protocols previously developed for other pear pathogenic *Erwinia* species, in order to check possible cross-reactions with *E*. *uzenensis*. Therefore, the three *E*. *uzenensis* strains were analyzed with real-time PCR protocols developed for *E*. *amylovora* by Gottsberger *et al*. [16] and Pirc *et al*. [17], for *E*. *pyrifoliae* by Wensing *et al*. [18], and for *E*. *piriflorinigrans* by Barbé *et al*. [3], using 10^6^ cfu ml^-1^ bacterial suspensions. Additionally, sensitivity and specificity assays of the developed primers and probe were performed in bacterial mixtures consisting of *E*. *uzenensis* and one of the other three pear pathogenic *Erwinia* species used in the specificity assays. Thus, there were three bacterial mixtures assayed, namely *E*. *uzenensis*:*E*. *amylovora*, *E*. *uzenensis*:*E*. *pyrifoliae*, and *E*. *uzenensis*:*E*. *piriflorinigrans*. The initial bacterial suspensions used for the preparation of the mixtures had a concentration of 10^6^ cfu ml^-1^, and were mixed in ratios 1:1, 1:2, 2:1 v/v. Same ratios for *E*. *uzenensis*:water were used as control. A second series of assays was conducted on similar mixtures prepared using initial bacterial suspensions with a concentration of 10^5^ cfu ml^-1^. All bacterial mixtures prepared were subjected to real-time PCR following the protocol described above. The experiments were performed in triplicate.

Sensitivity assays of the developed real-time PCR were performed on serial dilutions of bacterial suspensions and also on spiked samples of pear material. Briefly, a suspension of *E*. *uzenensis* LMG25844 strain was adjusted to 10^8^ cfu ml^-1^ at 600 nm (OD_600_ 0.2), and serial dilutions from 10^5^ to 10^1^ cfu ml^-1^ were prepared. A plate count on King’s medium B was used to confirm the bacterial concentration of each suspension. For spiked samples, 1 g of healthy pear tissue (twigs, flowers, buds, leaves, fruits) was slightly crushed with 50 ml of antioxidant maceration buffer, as described in the protocol of the European and Mediterranean Plant Protection Organization (EPPO) for *E*. *amylovora* [19] and then spiked with the serially diluted suspensions of *E*. *uzenensis* described above. In specific, 1 ml of the spiked sample consisted of 990 μl of macerated plant tissue in PBS and 10 μl of the bacterial suspension to reach a final concentration of 10^5^ to 10^1^ cfu ml^-1^.

The serially diluted bacterial suspensions were used directly in the real-time PCR reactions, while for the spiked samples, the DNA used was extracted following the protocol of Llop *et al*. [20]. In both cases, 5 μl of suspension or DNA were used per 25 μl reaction. The bacterial suspensions were prepared in 10 mM PBS in all the analyses. The negative control used included DNA extracted from healthy plant material macerated in PBS similarly to the spiked samples, while the no template control included only PBS.

The bacterial dilutions prepared for the sensitivity assays were employed as real-time PCR DNA standards to generate a standard curve of the fluorescence signal in relation to the amount of template. The threshold cycle values (Ct values) were plotted versus the known amount of bacteria.

## Results

### Cloning and sequencing of the *E*. *uzenensis* ribosomal operon

**Fig 4:**
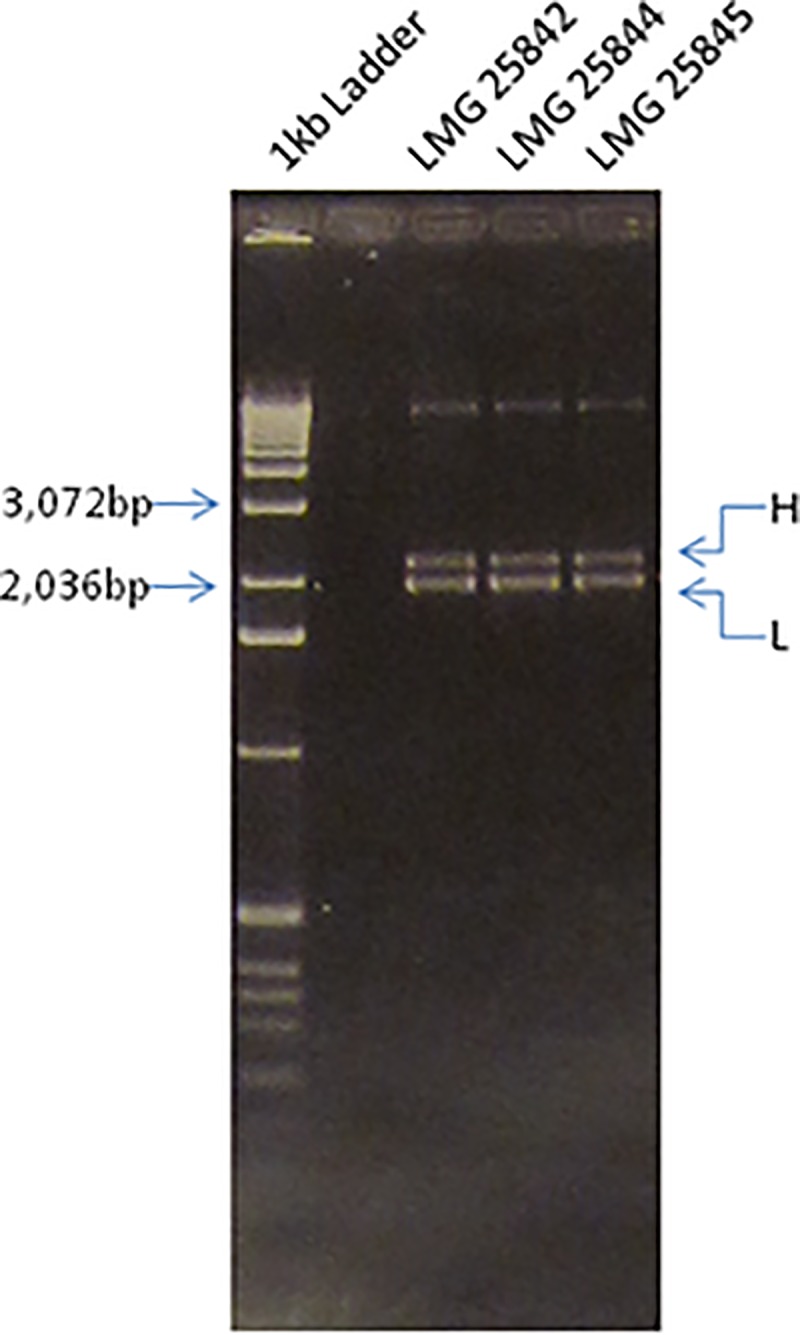
Agarose gel electrophoresis of the PCR products. Products generated by the primers FGPS6 and FGPL132’ on the three *E*. *uzenensis* strains: LMG 25842, LMG 25844, LMG 25845 are shown. Two distinct bands with a size ranging between 2,000 bp and 2,250 bp are present for all three *E*. *uzenensis* strains tested. The bands with higher or lower molecular weight are marked as ‘H’ or ‘L’, respectively, to the right of the gel. The 2036 bp and 3072 bp fragments of a 1 kb DNA ladder (Invitrogen, USA) loaded in the left side lane are also indicated.

Gel electrophoresis of the PCR products generated with primers FGPS6 and FGPL132' on the three *E*. *uzenensis* strains revealed the presence of two bands of ca. 2000 bp and 2250 bp for all of them. These bands were marked as ‘H’ or ‘L’, according to their higher or lower size, respectively (Fig 4). The presence of two bands in the PCR reactions suggests that the three isolates possess multiple rRNA operons with intragenomic ITS variation, which generally stems primarily from differences in tRNA gene composition, as already observed in other *Erwinia* species [21].

The two bands were cloned and sequenced for each of the three strains to deduce the information needed for the subsequent selection of the appropriate region for primers-probe design. Comparison of the derived sequences by the BLASTn algorithm (NCBI) confirmed that ‘L’ and ‘H’ bands correspond to sequences that are highly homologous to the 16S-ITS-23S rRNA region of other *Erwinia* strains, for example they are 98% identical over 99% of their length to that of *E*. *pyrifoliae* (GenBank Accession no: FN392235.1). The newly determined 16S-ITS-23S rRNA sequences of *E*. *uzenensis* have been deposited in the GenBank Database under accession numbers: KY314620 (strain LMG 25842, ‘H’), KY314621 (strain LMG 25842, ‘L’), KY314622 (strain LMG 25844, ‘H’), KY314623 (strain LMG 25844, ‘L’), KY314624 (strain LMG 25845, ‘H’) and KY314625 (strain LMG 25845, ‘L’).

Furthermore, using the RNAmmer and the tRNAscan-SE software [13, 14], the structural annotation of the *E*. *uzenensis* sequences obtained in this work was predicted with regard to the location of non-coding RNA sequences. For all three strains, a 16S rRNA gene was predicted to span a region of 1530 bp in the sequences corresponding to both the ‘L’ and ‘H’ bands. This 16S rRNA gene was ≥99% identical among the three *E*. *uzenensis* strains. However, in all three strains, the ‘L’ and ‘H’ bands differed in the organization pattern and size of their ITS region. In the case of ‘H’ bands, two tRNA molecule sequences were predicted in the ITS region: one tRNA^Ile^ at location 1611 bp to 1684 bp, and one tRNA^Ala^ at location 1841 bp to 1913 bp. In the case of ‘L’ bands, only one tRNA molecule sequence was predicted in the ITS region: a tRNA^Glu^ at location 1608 bp to 1680 bp. Finally, in all three strains and in both the ‘H’ and ‘L’ bands, the sequences included 128 bp of the 5’ end of the 23S rRNA gene (Fig 2).

### Real-time PCR development and determination of its specificity and sensitivity

**Fig 5:**
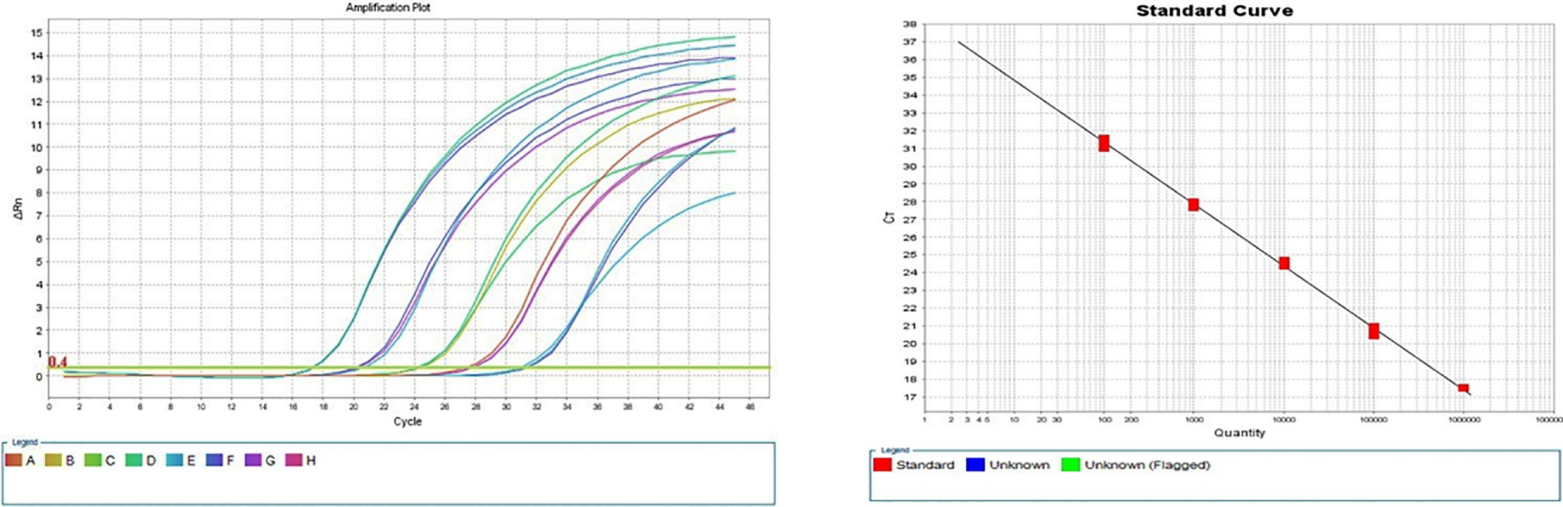
Amplification plot and standard curve analyses. Graphs obtained in the real-time PCR on E. uzenensis strain LMG25844 serially diluted (105 to 101 cfu ml-1) in 10 mM PBS. Linear correlation of the standard curve, R2: 0.996; Slope: -3.4; Efficiency: 96.6%.

The comparative analyses of the ITS consensus sequence of the *E*. *uzenensis* strains, as determined in the ‘H’ bands, with that of strains of related *Erwinia* species (*E*. *amylovora* and *E*. *pyrifoliae*) resulted in determining a 129 bp region in the ITS, suitable to design a pair of primers (UzeF, UzeR) and probe (UzeP) (Fig 3; Table 2). The developed real-time PCR protocol employing these primers and probe performed well when using a dilution series of culture suspension (10^5^ to 10^1^ cfu ml^-1^) of the *E*. *uzenensis* LMG25844 strain. Cycle threshold (Ct) values were linearly correlated with the quantity of the target sample and permitted the construction of the standard curve (Fig 5). The correlation coefficient (R^2^) was 0.996, with a slope of -3,4 and efficiency of 96,6%.

Different assays were performed to check both specificity and sensitivity of the developed real-time PCR protocol. Specificity was firstly analyzed *in silico* by BLASTn searches of the sequences of the primers and probe designed in this work. These analyses showed that whereas primer UzeR hybridized with several locations within the *E*. *amylovora* ITS (100% identity in 19 out of the 20 nts), primer UzeF and probe UzeP did not give enough matches against sequences of the known, closely related pear pathogens. This supports the reliability of the PCR developed by *in silico* analyses. Additionally, when the developed real-time PCR protocol was applied on all pathogenic and non-pathogenic *Erwinia* strains included in Table 1, only the *E*. *uzenensis* strains provided a positive result

Furthermore, *E*. *uzenensis* strains did not provide positive results with any of the real-time PCR protocols developed to detect the other pathogenic *Erwinia* species. The sensitivity of the developed PCR using the bacterial suspensions of *E*. *uzenensis* reached the level of 10^1^ cfu ml^-1^, and with the spiked pear samples, the level of 10^2^−10^4^ cfu ml^-1^ depending on the plant material analyzed (Table 3). The sensitivity of the real-time PCR was not affected in the presence of other *Erwinia* species, with the same Ct values as the *E*. *uzenensis*:water control, even in ratios of 1:2 (data not shown). The real-time PCR was positive only when *E*. *uzenensis* was included in the mix, and negative when *E*. *amylovora*, *E*. *pyrifoliae* or *E*. *piriflorinigrans* were tested alone.

**Table 3:** Ct values obtained in sensitivity assays using spiked pear samples consisting of various types of tissue and serial dilutions of *E*. *uzenensis* to reach a final bacterial concentration ranging from10^5^ to 10^1^ cfu ml^-1^.

Plant material		Ct values* obtained at a concentration of *E*. *uzenensis* in the spiked samples				
Host	Tissue	10^5^ cfu ml^-1^	10^4^ cfu ml^-1^	10^3^ cfu ml^-1^	10^2^ cfu ml^-1^	10^1^ cfu ml^-1^
*Pyrus* sp.	flowers	19.5± 0.10	22.4±0.08	25.8±0.15	31.5±0.07	-**
	buds	23.3±0.22	26.7±0.10	30.7±0.09	-	-
	leaves	20.0±0.08	23.6±0.05	27.6±0.30	30.8±0.21	-
	fruits	23.4±0.13	27.0±0.10	-	-	-
	twigs	20.5±0.06	22.9±0.09	26.2±0.15	29.1±0.07	-

* Ct values: mean of the Ct values of three replicates ± standard deviation of these values

** ‘-’negative result

## Discussion

Some pear pathogenic *Erwinia* species constitute a serious threat for the cultivation of this economically-important crop, as well as for other rosaceous fruit trees (apple, quince, loquat, etc.) and ornamental species of economic value. *E*. *amylovora* is the most destructive species of this genus, considering its large host range [19] and the severity of symptoms caused [7], resulting in significant crop losses [7]. However, other pathogenic *Erwinia* species may contribute to significant losses on these crops, as well. This is supported by certain features of these *Erwinia* pathogens: a) the presence of plasmids genetically similar to the ubiquitous, virulence-related plasmid pEA29 of *E*. *amylovora*, such as the plasmid pEJ30 of *Erwinia* sp. strains causing bacterial shoot blight on pear in Japan [22] and the plasmid pEPIR37 of *E*. *piriflorinigrans* causing pear blossom necrosis, which has been proven to increase symptom development when introduced in a *E*. *amylovora* strain cured of pEA29 [23]; b) their possibly underestimated occurrence, due to the lack of reliable diagnostic methods, as suggested by the recent records showing the occurrence of *E*. *pyrifoliae* and *E*. *piriflorinigrans* in new geographic areas and new hosts [3, 4], (Smits, T. personal communication); c) the presence of transferable plasmids in these pathogens [24] which suggests that interchange of genetic information through horizontal gene transfer events is possible, and lastly d) the occurrence of mixed infections on host plants, as shown for *E*. *amylovora* and *E*. *piriflorinigrans* which were isolated jointly from loquat plants outbreaks in Spain [3] indicating a possible synergistic effect in enhancing symptom development. These features substantiate the need for specific and sensitive detection protocols for all pathogenic *Erwinia* species, that can provide: a) reliable identification of the different pathogens affecting a host, especially when the possible causal agents are so closely related genetically and their disease symptoms can be misidentified due to their similarity; b) further insight into the epidemiological behavior of each species, alone, or in combination of two or more of them, as well as the real host range of each species. The need for such detection protocol is obvious for *E*. *uzenensis*, a newly-described pear pathogenic *Erwinia* species for which not much information is available about its geographical distribution, host range, and life cycle. To address this knowledge gap, a new molecular detection method, based on a real-time PCR protocol using a specific probe, has been developed in this work, showing reliability in specificity and sensitivity, even with mixed related bacteria in the same sample.

Because little genetic information is available on *E*. *uzenensis*, its ribosomal operon was sequenced and the ITS region was selected to design primers and probe for real-time PCR with high specificity. Three *E*. *uzenensis* strains were used in the study, as the number of such strains available in the bacterial collections worldwide is very limited. The comparisons performed on the *E*. *uzenensis* ITS consensus sequence against the respective region of the other *Erwinia* pathogenic species allowed the selection of a region sufficiently variable to design a pair of primers and a TaqMan probe with good specificity against closely related pear-associated *Erwinia* species. The developed real-time PCR protocol was evaluated using bacterial cell suspensions and plant material artificially spiked with *E*. *uzenensis*. Due to the very restricted geographical distribution of this pathogen (recorded only in Japan), plant tissue naturally infected by this pathogen was not available to the authors for further evaluation of the protocol. Nevertheless, the results obtained showed that the developed real-time PCR protocol is specific in not detecting any of the other closely related *Erwinia* species tested and shows a good sensitivity level in detecting *E*. *uzenensis* in cell suspensions and in spiked plant material. The high sensitivity obtained could be explained by the presence of DNA from dead cells that would increase the template available for amplification by PCR not reflected in the counting by plating, as reported in previous works [25]. The differences in the sensitivity level obtained with the various types of plant material used could be due to the dissimilar efficiency of the DNA extraction protocol to remove the inhibitors present in variable amounts in fruit, leaf, bark and stem tissues. Similarly to the detection methods recently developed for other newly-described pathogenic *Erwinia* species [3, 4], this protocol would provide an important tool for diagnostic and epidemiological studies, expanding our knowledge on this new species.

As the symptoms caused by *E*. *uzenensis* on pear can be misidentified as being caused by *E*. *amylovora* or other *Erwinia* species, it is not recommended to make a diagnosis on the basis of symptoms observation. Furthermore, the identification of *E*. *uzenensis* is confusing because it shares phenotypic characteristics with other pathogenic and non-pathogenic *Erwinia* species. The real-time PCR protocol presented here accurately identifies *E*. *uzenensis* in spiked plant material and could be used in surveys investigating the current distribution of this pathogen and in epidemiological studies that would provide information on its life cycle in the plant tissues.

## References

[1] Mizuno A, Tsukamoto T, Shimizu Y, Ooya H, Matsuura T, Saito N, et al Occurrence of bacterial black shoot disease of European pear in Yamagata Prefecture. J General Plant Pathol 2010;76: 43–51.

[2] Matsuura T, Mizuno A, Tsukamoto T, Shimizu Y, Saito N, Sato S, et al *Erwinia uzenensis* sp. *nov*., a novel pathogen that affects European pear trees (*Pyrus communis* L.). Int J Syst Evol Microbiol. 2012;62: 1799–1803. 10.1099/ijs.0.032011-0 21986725

[3] Barbé S, Bertolini E, Roselló M, Llop P, López MM. Conventional and real-time PCRs for detection of *Erwinia piriflorinigrans* allow its distinction from the fire blight pathogen, *Erwinia amylovora*. Appl Environ Microbiol. 2014;80: 2390–2398. 10.1128/AEM.03626-13 24509928 PMC3993200

[4] Wenneker M, Bergsma-Vlami M. *Erwinia pyrifoliae*, a new pathogen on strawberry in the Netherlands. J Berry Res. 2015;5: 17–22.

[5] Rezzonico F, Smits TH, Born Y, Blom J, Frey JE, Goesmann A, et al *Erwinia gerundensis* sp. nov., a cosmopolitan epiphyte originally isolated from pome fruit trees. Int J Syst Evol Microbiol. 2016;66: 1583–1592. 10.1099/ijsem.0.000920 26813696

[6] Council Directive 2000/29/EC. Official Journal of the European Communities. L 169. 112 pp.

[7] EFSA Panel on Plant Health (PLH). Scientific Opinion on the pest categorization of *Erwinia amylovora* (Burr.) Winsl. et al EFSA Journal. 2014;12: 3922–3958.

[8] King EO, Ward M, Raney DE. Two simple media for the demonstration of pyocyanin and fluorescein. J Lab Clin Med. 1954;44: 401–407. 13184240

[9] Sambrook J, Fritsch EF, Maniatis T. Molecular cloning: a laboratory manual 2nd ed. Cold Spring Harbor Laboratory, Cold Spring Harbor, NY; 1989.

[10] Ponsonnet C, Nesme X. Identification of *Agrobacterium* strains by PCR-RFLP analysis of pTi and chromosomal regions. Arch Microbiol. 1994;161: 300–309. 7911654 10.1007/BF00303584

[11] Osborn AM, Moore ERB, Timmis KN. An evaluation of terminal-restriction fragment length polymorphism (T-RFLP) analysis for the study of microbial community structure and dynamics. Environ Microbiol. 2000;2: 39–50. 11243261 10.1046/j.1462-2920.2000.00081.x

[12] Nakagawa T, Shimada M, Mukai H, Asada K, Kato I, Fujino K, et al Detection of alcohol-tolerant hiochi bacteria by PCR. Appl Environ Microbiol. 1994;60: 637–640. 7510942 10.1128/aem.60.2.637-640.1994 PMC201360

[13] Lagesen K, Hallin PF, Rødland E, Stærfeldt HH, Rognes T, Ussery DW. RNammer: consistent annotation of rRNA genes in genomic sequences. Nucleic Acids Res. 2007;35: 3100–3108. 10.1093/nar/gkm160 17452365 PMC1888812

[14] Schattner P, Brooks AN, Lowe TM. The tRNAscan-SE, snoscan and snoGPS web servers for the detection of tRNAs and snoRNAs. Nucleic Acids Res. 2005;33: 686–689. 10.1093/nar/gki366 PMC1160127 15980563

[15] Thompson JD, Higgins DG, Gibson TJ. Clustal-W–Improving the sensitivity of progressive multiple sequence alignment through sequence weighting, position-specific gap penalties and weight matrix choice. Nucleic Acids Res 1994;22: 4673–4680. 10.1093/nar/22.22.4673 7984417 PMC308517

[16] Gottsberger RA. Development and evaluation of a real-time PCR assay targeting chromosomal DNA of *Erwinia amylovora*. Letters Appl Microbiol. 2010;51: 285–292. 10.1111/j.1472-765X.2010.02892.x 20666990

[17] Pirc M, Ravnikar M, Tomlinson J, Dreo T. Improved fireblight diagnostics using quantitative real-time PCR detection of *Erwinia amylovora* chromosomal DNA. Plant Pathol. 2009;58: 872–881.

[18] Wensing A, Gernold M, Geider K. Detection of *Erwinia* species from the apple and pear flora by mass spectroscopy of whole cells and with novel PCR primers. J Applied Microbiol. 2012;112: 147–158. 21973322 10.1111/j.1365-2672.2011.05165.x

[19] European and Mediterranean Plant Protection Organization (EPPO) Diagnostic. PM 7/20 (2) *Erwinia amylovora*. Bulletin OEPP/ EPPO Bulletin. 2013;43: 21–45.

[20] Llop P, Caruso P, Cubero J, Morente C, López MM. A simple extraction procedure for efficient routine detection of pathogenic bacteria in plant material by polymerase chain reaction. J. Microbiol Methods. 1999;37: 23–31. 10395461 10.1016/s0167-7012(99)00033-0

[21] Stewart FJ, Cavanaugh CM. Intragenomic variation and evolution of the internal transcribed spacer of the rRNA operon in bacteria. J Mol Evol. 2007;65: 44–67. 10.1007/s00239-006-0235-3 17568983

[22] Maxson-Stein K, McGhee GC, Smith JJ, Jones AL, Sundin GW. Genetic analysis of a pathogenic *Erwinia* sp. isolated from pear in Japan. Phytopathology. 2003;93: 1393–1399. 10.1094/PHYTO.2003.93.11.1393 18944067

[23] Barbé S, Llop P, Blom J, Cabrefiga J, Goesmann A, Duffy et al Complete sequence of *Erwinia piriflorinigrans* plasmids pEPIR37 and pEPIR5 and role of pEPIR37 in pathogen virulence. Plant Pathol. 2013;62: 786–798.

[24] Llop P, Barbé S, López MM. Functions and origin of plasmids in *Erwinia* species that are pathogenic to or epiphytically associated with pome fruit trees. Trees. 2012;26: 31–46. 10.1007/s00468-011-0630-2 25983394 PMC4425259

[25] Llop P, Bonnaterra A, Peñalver J, López MM. Development of a highly sensitive nested-PCR procedure using a single closed tube for detection of *Erwinia amylovora* in asymptomatic plant material. Appl Environ Microbiol. 2000;66: 2071–2078. 10.1128/aem.66.5.2071-2078.2000 10788384 PMC101457

[26] Paulin JP, Samson R. Le feu bactérien en France. II. Caractères des souches d'*Erwinia amylovora* (Burrill) Winslow *et al*, 1920, isolées du foyer franco-belge. Annal Phytopathol. 1973;5: 389–397.

[27] Llop P, López MM, Cabrefiga J, Ruz L, Montesinos E. Study of the virulence in wild-type strains of *Erwinia amylovora* devoid of the plasmid pEa29. Acta Hort. 2008;793: 145–148.

[28] Llop P, Cabrefiga J., Smits TH, Dreo T, Barbé S, Pulawska J, et al *Erwinia amylovora* novel plasmid pEI70: complete sequence, biogeography, and role in aggressiveness in the fire blight phytopathogen. PLoS ONE. 2011;6 (12) e28651 10.1371/journal.pone.0028651 22174857 PMC3235134

[29] Brennan JM, Doohan FM, Egan D, Scanlan H, Hayes D. Characterization and differentiation of Irish *Erwinia amylovora* isolates. J Phytopathology. 2002;150: 414–422.

[30] Cesbron S, Paulin JP, Tharaud M, Barny MA, Brisset MN. The alternative sigma factor HrpL negatively modulates the flagellar system in the phytopathogenic bacterium *Erwinia amylovora* under *hrp*-inducing conditions. FEMS Microbiol Lett. 2006;257: 221–227. 10.1111/j.1574-6968.2006.00172.x 16553857

[31] Rezzonico F, Smits TH, Duffy B. Diversity, evolution, and functionality of clustered regularly interspaced short palindromic repeat (CRISPR) regions in the fireblight pathogen *Erwinia amylovora*. Appl Environ Microbiol. 2011;77: 3819–3829. 10.1128/AEM.00177-11 21460108 PMC3127596

[32] Kim W-S, Gardan L, Geider K, Rhim SL. *Erwinia pyrifoliae* sp. nov., a novel pathogen affecting Asian pear trees (*Pyrus pyrifolia* Nakai). Int J Syst Bacteriol. 1999;49: 899–906 10.1099/00207713-49-2-899 10319516

[33] López MM, Roselló M, Llop P, Ferrer S, Christen R, Gardan L. *Erwinia piriflorinigrans* sp. nov., a novel pathogen that causes necrosis of pear blossoms. Int J Syst Evol Microbiol. 2011;61: 561–567. 10.1099/ijs.0.020479-0 20382791

[34] Geider K, Auling G, Du Z, Jakovljevic V, Jock S, Völksch B. *Erwinia tasmaniensis* sp. nov., a non phytopathogenic bacterium from apple and pear trees. Int J Syst Evol Microbiol. 2006;56: 2937–2943. 10.1099/ijs.0.64032-0 17159002

[35] Mergaert J, Hauben L, Cnockaert MC, Swings J. Reclassification of non-pigmented *Erwinia herbicola* strains from trees as *Erwinia billingiae* sp. *nov*. Int J Syst Evol Microbiol. 1999;49: 377–383. 10.1099/00207713-49-2-377 10319458

